# Multifactorial Etiology of Anemia in Celiac Disease and Effect of Gluten-Free Diet: A Comprehensive Review

**DOI:** 10.3390/nu11112557

**Published:** 2019-10-23

**Authors:** Rafael Martín-Masot, Maria Teresa Nestares, Javier Diaz-Castro, Inmaculada López-Aliaga, Maria Jose Muñoz Alférez, Jorge Moreno-Fernandez, José Maldonado

**Affiliations:** 1 Pediatric Clinical Management Unit, Málaga Regional University Hospital, 29010 Málaga, Spain; rafammgr@gmail.com; 2 Department of Physiology and Institute of Nutrition and Food Technology “José MataixVerdú”, University of Granada, 18071 Granada, Spain; javierdc@ugr.es (J.D.-C.); milopez@ugr.es (I.L.-A.); malferez@ugr.es (M.J.M.A.); jorgemf@ugr.es (J.M.-F.); 3 Department of Pediatrics, University of Granada, 18071 Granada, Spain; jmaldon@ugr.es; 4 Biosanitary Research Institute, 18012 Granada, Spain; 5 Maternal and Child Health Network, Institute Carlos III, 28020 Madrid, Spain; 6 Pediatric Clinical Management Unit. Virgen de las Nieves University Hospital, 18014 Granada, Spain

**Keywords:** celiac disease, gluten-free diet, iron deficiency, anemia, micronutrient deficiencies

## Abstract

Celiac disease (CD) is a multisystemic disorder with different clinical expressions, from malabsorption with diarrhea, anemia, and nutritional compromise to extraintestinal manifestations. Anemia might be the only clinical expression of the disease, and iron deficiency anemia is considered one of the most frequent extraintestinal clinical manifestations of CD. Therefore, CD should be suspected in the presence of anemia without a known etiology. Assessment of tissue anti-transglutaminase and anti-endomysial antibodies are indicated in these cases and, if positive, digestive endoscopy and intestinal biopsy should be performed. Anemia in CD has a multifactorial pathogenesis and, although it is frequently a consequence of iron deficiency, it can be caused by deficiencies of folate or vitamin B_12_, or by blood loss or by its association with inflammatory bowel disease (IBD) or other associated diseases. The association between CD and IBD should be considered during anemia treatment in patients with IBD, because the similarity of symptoms could delay the diagnosis. Vitamin B_12_ deficiency is common in CD and may be responsible for anemia and peripheral myeloneuropathy. Folate deficiency is a well-known cause of anemia in adults, but there is little information in children with CD; it is still unknown if anemia is a symptom of the most typical CD in adult patients either by predisposition due to the fact of age or because biochemical and clinical manifestations take longer to appear.

## 1. Introduction

Celiac disease (CD) is one of the most frequent genetic diseases, affecting 1% of the world population. Diagnosed cases are increasing and it seems to be due to the actual increase in the incidence rather than due to the advancement of diagnostic methods or to the larger awareness of the disease among the lay population [1,2].

Celiac disease is a systemic disorder, caused by an immune reaction activated by the ingestion of gluten and related proteins occurring in individuals carrying haplotypes of major histocompatibility antigen (HLA) class II: more than 90% of celiac patients are HLA-DQ2 haplotype positive, and almost all of the remaining patients carry HLA-DQ8. Exposure to gluten has a double effect, triggering both innate and adaptive immune responses, with symptoms at the intestinal and extra-intestinal levels [3]. The contact of the intestinal mucosa with gluten leads to a characteristic histological lesion, although not pathognomonic. Their typical histological features are an increase in intraepithelial lymphocytes, villous atrophy, crypt hyperplasia, and infiltration of inflammatory cells in the lamina propria.

Diagnosis of CD is conducted by combining serological screening tests (anti-tissue-transglutaminase and anti-endomysial IgA antibodies) and an intestinal biopsy [4]. The duodenal biopsy can be avoided [5] in adolescents and children with symptoms or signs of CD and with high anti-tissue-transglutaminase antibody levels, positivity for anti-endomysial antibodies, and presence of HLA DQ21 or HLA-DQ8 heterodimer.

Recent reports have demonstrated that specific miRNAs are modulated in duodenal mucosa affected by CD. The miRNAs dysregulated during the development of CD could be potentially involved in the pathogenesis of CD [6]. Overexpression or downregulation of several miRNAs could potentially stimulate or inhibit pathways related to the pathogenesis of CD. A study has demonstrated the regulation of circulating miRNA-21 and miRNA-31 expression levels in children with CD and showed that miR-21 expression level was positively correlated with the anti-tissue-transglutaminase IgA antibodies [7]. This correlation may indicate that the altered expression of the circulating miRNAs could be used as potential non-invasive diagnostic and prognostic biomarkers for CD patients. In addition, Vaira et al. [8] have shown the downregulation of miR-194-5p and the overexpression of miR-638 in celiac patients with anemia compared with celiac patients with classical symptoms.

Patients with CD could feature various deficiency states, leading to anemia and bone mass loss and a wide range of digestive and extra-digestive symptoms. Upon diagnosis, nutritional deficiencies were found in vitamins and minerals; patients should be tested for micronutrient deficiencies, in particular iron, folic acid, vitamin B_12_, vitamin D, copper, and zinc. Celiac disease is a cause of anemia, usually due to the malabsorption of iron, folic acid, and vitamin B_12_ [9]. Anemia is mainly due to the fact of iron deficiency as a consequence of iron malabsorption. Iron malabsorption is usually observed in CD, being considered a clinical diagnostic feature of CD even in subjects not presenting the classic digestive symptoms. Iron deficiency anemia (IDA) is a frequent finding in patients with overt CD (10–20% of cases) [10], despite the fact that they are consuming iron supplements. A recent meta-analysis found that more than 3% of patients with IDA have histological evidence of CD. This high percentage of subjects with IDA who are celiac, reinforces the need for screening CD in patients with IDA [11]. Folate and vitamin B_12_ malabsorption, nutritional deficiencies, blood loss, inflammation, development of refractory CD or concomitant *Helicobacter pylori* infection are other causes of anemia in such patients [12] (Table 1).

The mainstay of treatment for CD remains adherence to a gluten-free diet (GFD). In the vast majority of cases, strict monitoring of GFD leads to the disappearance of clinical symptoms and serological signs, the recovery of normal histology in the duodenum and the prevention of complications derived from CD [13]. However, in approximately 20% of celiac patients, symptoms persist despite excluding gluten from their diet [14].

The aim was to perform a review of recent literature data regarding causes of anemia in CD patients. For this purpose, we performed a literature search on two databases—PubMed and Embase—using the Medical Subject Headings (MESH) term “celiac disease” and several keywords referring to the associated hematological features and nutritional imbalances. Articles identified from this search strategy were evaluated for relevance to the topic. Clinically significant full-text articles were selected for their inclusion in this review.

## 2. Micronutrient Deficiencies and Celiac Disease

### 2.1. Iron Deficiency

Iron is an essential micronutrient, it is required for adequate erythropoietic function, oxidative metabolism, enzymatic activities, and cellular immune responses [15]. IDA is a major public health problem. Iron deficiency anemia occurs when iron loss and body’s requirement for iron are not met by dietary sources, therefore the iron storage of the organism is depleted. This pathological process is characterized by the production of smaller red cells because the concentration of hemoglobin (Hb) is abnormally low [15]. Iron deficiency anemia results in fatigue and diminished muscular oxygenation, which may affect muscle strength and quality and, subsequently, physical performance [16]. Celiac disease constitutes one of the groups at highest risk of iron deficiency (ID) [17]. Iron requirements exceed iron intake at some time points throughout life: the first 6–18 months of life and then, for women, during adolescence and all fertile period. Iron deficiency during the first year of life occurs at a time point of rapid neural development and when morphological, biochemical, and bioenergetic alterations may all influence future functioning [18]. The brain is the most vulnerable organ during critical periods of development [19]. Iron is present in the brain from very early in life, when it participates in the neural myelination processes [20], learning, and interacting behaviors, and iron is needed by enzymes involved in the synthesis of serotonin and dopamine neurotransmitters [21].

The most common causes of ID are blood loss and failure of the enterocytes of the proximal intestine to uptake iron from the diet in patients who have enough dietary iron. Celiac disease leads to an abnormal immune response, which is followed by a chronic inflammation of the small intestinal mucosa with progressive disappearance of intestinal villi [22] leading to a decrease in absorption of many nutrients, including iron [23,24]. Unfortunately, this interesting association between CD and IDA has been poorly appreciated [25] in spite of the great interest of micronutrient deficiency as a diagnostic clue in asymptomatic CD, especially for iron and IDA [26].

Celiac disease is an increasingly recognized disorder in Caucasian populations of European origin. Murray et al. [27] analyzed HLA genotypes and frequencies of CD between Caucasians and non-Caucasians with ID. The results showed that CD is associated with ID in Caucasians, but CD is rare among non-Caucasians—even among individuals with features of CD, such as ID. Pirán Arce et al. [28] evaluated the nutritional status of iron in 44 celiac children by determining biochemical parameters and their relationship with the intake of this mineral and adherence to the GFD. These authors concluded that under conditions of adequate iron consumption, iron status is related to the degree of adherence to the GFD. Although GFD is an effective treatment for CD, IDA remains an occasional finding during follow-up and correlates to inadequate gluten exclusion [10].

Malabsorption causes should be considered especially in refractory IDA; this malabsorption can be the only manifestation in subclinical and silent CD [29,30]. The study of Shahriari et al. [11] suggests serologic screening for CD in patients with refractory IDA to minimize the complications of CD and repeated iron treatment. A study [31] revealed a significant association between *H. pylori* infection and IDA in patients with CD, and Samasca et al. [32] recommend performing the screening for *H. pylori* infection in patients with CD and ID, but currently there is no evidence to support this recommendation.

Elli et al. [33] evaluated the role of the *TMPRSS6* variant *rs855791* in GFD treated CD patients with IDA persistence against non-IDA CD and non-CD subjects. The authors found a significantly higher percentage of *TMPTSS6* mutation in CD patients than in non-CD controls, while no differences were found between IDA and non-IDA CD patients. Conversely, De Falco et al. [34] investigated the role of HFE *C282Y*, *H63D*, and *TMPRSS6 A736V* gene variants in the pathogenesis of IDA in CD patients, at diagnosis and after 1 year of GFD. This study suggests a protective role of HFE in IDA CD patients and confirms the role of *TMPRSS6* in predicting oral iron response modulating hepcidin action on iron absorption. Iron supplementation therapeutic management in CD could depend on *TMPRSS6* genotype that could predict persistent IDA despite iron supplementation and GFD.

**Figure 1:**
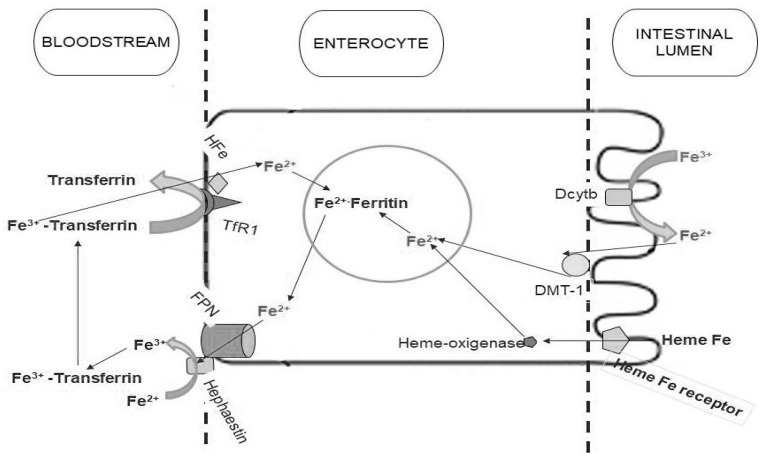
Iron absorption metabolism. Non-heme iron is ultimately taken up from the lumen by divalent metal transporter (DMT-1) on the microvillus membrane, before joining the labile iron pool in the cell. Ferric iron has to be reduced to the ferrous form by duodenal cytochrome b (Dcytb) before the uptake. Ferrous iron in the labile iron pool is then transferred to the circulation by ferroportin (FPN), which requires hephaestin for oxidation to the ferric form to bind transferrin. Heme iron is taken up by a specific receptor. Internalized heme iron is degraded by heme-oxygenase, releasing non-heme iron. The non-heme iron is then transported to the cytoplasm, joining the labile iron pool and is then transferred to the bloodstream by FPN in the same manner as non-heme iron.

Iron enters the enterocytes through an apical divalent metal transporter (DMT-1) (Figure 1). Sharma et al. [35] have evaluated iron regulatory proteins in celiac patients compared to controls and iron deficient patients using duodenal biopsies. The results showed that DMT-1, ferroportin, hephaestin, and transferrin receptor protein mRNA increased, primarily due to the fact of iron deficiency, while body iron stores were reduced in CD. In contrast, these authors [35] showed that expression of DMT1 and ferroportin are increased in CD patients with or without ID. In this study, ferritin expression was also found to be increased in CD, but only in those with ID.

Tolone et al. [36] reported the link between DMT-1 *IVS4+44C-AA* and anemia in 387 Italian celiac children and the functional role of the polymorphism. They found that the DMT-1 *IVS4+44-AA* genotype confers a four-fold risk of developing anemia, despite the atrophy degree. Anemia in patients with CD is multifactorial.

Patients with CD may benefit from iron supplementation (iron sulfate), but intolerance to iron sulfate could reduce the efficacy of this supplementation. Sucrosomial iron, a presentation of ferric pyrophosphate covered by a phospholipid and sucrester membrane, can be effective in providing iron supplementation in difficult-to-treat patients with CD and intolerance to iron sulfate, allowing good intestinal absorption independently of the DMT-1 carrier [37]. A study provides evidence that Feralgine^TM^, a solution of ferrous bisglycinate chelate and sodium alginate, is well absorbed in celiac patients [38]. Furthermore, it might by suggested that the iron complex might be absorbed regardless of the presence of DMT-1.

**Figure 2:**
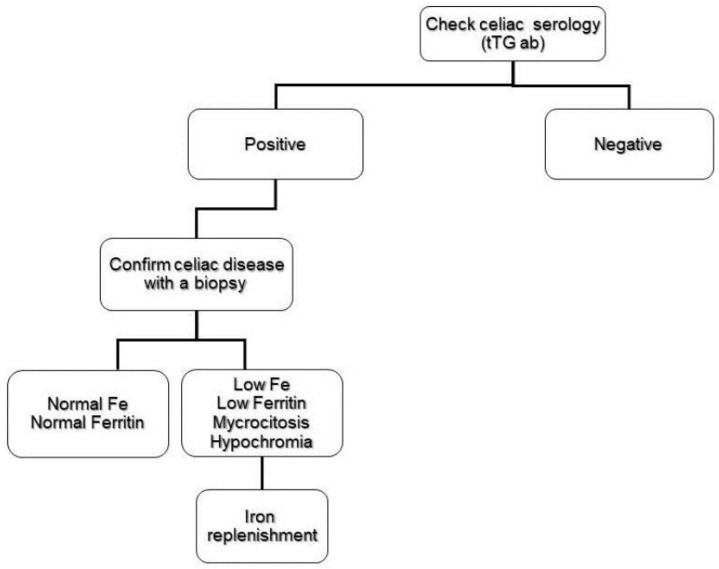
Abbreviated flow chart of the investigation of iron deficiency anemia in celiac disease patients.

The prevalence of CD in subjects presenting IDA has been described by other authors [39,40,41] with different results, due to the probable differences in the study of the designs. Lasa et al. [40] designed a study to avoid the abovementioned bias. They decided to evaluate all patients diagnosed with IDA by performing upper endoscopy and duodenal biopsies, and not only those with positive antibodies or with IDA of unknown origin (after an extensive work-up). Patients with IDA have an increased risk for CD, up to 25% of these patients may not present any endoscopic sign suggesting villous atrophy [39]. This finding makes routine duodenal biopsy necessary when performing upper endoscopy on IDA patients. In a systematic review and meta-analysis, Mahadev et al. [3] found that approximately 1 out of 31 patients with IDA have histologic evidence of CD; this prevalence value justifies the screening of patients with IDA for CD (Figure 2).

### 2.2. Folate and Vitamin B_12_ Deficiency

Usually, people suffering from CD can develop folate and vitamin B_12_ deficiencies as a result of generalized malabsorption linked to villi atrophy. Both vitamins are essential for normal hematopoiesis and neurologic function.

Folate absorption occurs primarily in the jejunum, which is commonly affected by CD [10,42]. Several studies in adult celiac patients have shown an increased risk of folate deficiency, which can reach up to 20–30% of newly diagnosed patients [43,44]. Prior to uptake, folate must be deconjugated by a brush border membrane peptidase and the intestinal mucosa damage in CD may affect enzyme activity leading to a folate deficiency. Serum and red cell folate measurements are usually used for the diagnosis of folate deficiency. Serum folate levels reflect largely folate intake and it is common for levels to be high in patients with a vitamin B_12_ deficiency. Red cell folate is not a specific indicator for folate deficiency, as it can be decreased in patients with vitamin B_12_, but red cell folate levels are less influenced by variations in folate intake. Patients with CD commonly have elevated levels of homocysteine which may serve as an important clue for the diagnosis. However, the sensitivity of this measurement is somewhat less for vitamin B_12_ deficiency [45].

Vitamin B_12_ requires formation of a primary complex with intrinsic factor to be absorbed in the proximal small intestine, and small amounts may also be absorbed by passive transport throughout the entire intestine. Deficiency of vitamin B_12_ is common in CD and frequently results in anemia. Though the terminal ileum is the primary site of absorption of vitamin B_12_, García-Manzanares and Lucendo [44] reported a prevalence of vitamin B_12_ deficiency between 8% and 41% in patients with newly diagnosed CD.

The causes of B_12_ deficiency in CD are still not clear, but they may be related to complications of small intestinal injury including a decreased gastric acidity, cobalamin intake due to the frequent finding of bacterial overgrowth, autoimmune gastritis, and decreased efficiency of the intrinsic factor or even dysfunction of the distal small intestine. Abnormalities in the absorption of folate or vitamin B_12_ may result in anemia in children with untreated CD. The range of low folate and low vitamin B_12_ prevalence were 15.7–18.3% and 4.3–8%, respectively [42,46].

Both folate and vitamin B_12_ deficiencies can lead to a macrocytic anemia with low values for hemoglobin or hematocrit, and high mean corpuscular volume levels. Vitamin B_12_ deficiency should be considered in patients with CD and hematological and neurological disorders [47]. Vitamin B_12_ levels measured within the lower range of normal or if they coexist with folic acid deficiency can be misleading and difficult to interpret. Under these circumstances, high serum levels of methylmalonic acid may improve the diagnostic accuracy of vitamin B_12_ deficiency [48].

### 2.3. Copper and Zinc Deficiency

Micronutrient deficiencies are common in celiac patients. In addition to the abovementioned deficiencies (i.e., iron, folic acid, and vitamin B_12_), at the time of diagnosis there may be deficiencies for other vitamins and minerals, in particular copper and zinc [22].

Copper deficiency is a rare complication in CD and its prevalence remains unknown. This deficiency can lead to anemia, thrombocytopenia, neutropenia, and peripheral neuronal involvement. In adult celiac patients, peripheral myeloneuropathy has been described along with hypocupremia with a good clinical response to copper supplementation [49,50]. Halfdanarion et al. [51] reported five cases of adult celiac patients with copper deficiency; all of them presented neurological complications and three of them presented hematological abnormalities. Cavallieri et al. [52] recently described a rare case of myelopathy induced by copper deficiency secondary to undiagnosed CD, and they have suggested that patients with hypocupremia should be tested for CD.

Likewise, the presence of clinical alterations as a consequence of zinc deficiency is also uncommon in celiac patients. Fractional zinc absorption is no different between celiac patients and controls, but the rapid zinc exchange body compartment is lower in CD than in control patients [49]. The mechanism of zinc depletion and its possible implications are unknown [53].

## 3. Aplastic Anemia and Celiac Disease

Celiac disease has been linked to various hematological abnormalities [54], such as anemia, thrombopenia or thrombocytosis, leukopenia, splenic dysfunction, immunoglobulin A deficiency or lymphoma. Anemia is the most frequent cause of CD. In addition to the various etiologies of anemia in CD (iron deficiency, due to the micronutrient deficiency or chronic disorders), various cases of aplastic anemia associated with CD have been described in the literature [55,56,57,58,59,60], both in pediatric age and in adulthood. Despite the underlying mechanism of this association being still unknown [55], it has been suggested that both conditions might share a similar underlying pathophysiological mechanism, mediated by autoreactive T cells involved in tissue destruction [56] (Table 2). In all cases, the patient presented with pancytopenia and the diagnosis was achieved by bone marrow biopsy. Pancytopenia was resolved with the GFD only in some cases [57], while in other cases, the response was only partial and immunosuppressive treatment was required or even hematopoietic progenitor transplantation. Although infrequent, aplastic anemia may be an underdiagnosed entity [56], so it will be necessary to have the diagnostic suspicion both in the case of pancytopenia without apparent cause and in CD with pancytopenia. The etiology of anemia may be due to the presence of several factors, such as autoimmunity or chronic inflammation caused by the CD [60]. Based on the cases reported to date, it seems that the GFD is not enough to improve pancytopenia; therefore, most patients require other treatments. Some authors have suggested that the prognosis is better at pediatric age, possibly because the duration of exposure to chronic inflammation is shorter, and the GFD may probably reverse the process [57].

## 4. Anemia of Chronic Disease

Anemia of chronic disease (ACD) is an old concept in the scientific literature, but current research on the role of pro-inflammatory cytokines and iron metabolism has yielded more information about the pathophysiology of this disease. This type of anemia is linked to the deterioration of the production of erythrocytes associated with chronic inflammatory conditions including cancer, infections or autoimmune diseases. In addition, recent epidemiological studies have linked ACD with obesity, aging, and kidney failure. This type of anemia responds to a multifactorial pathogenesis including four fundamental mechanisms. These mechanisms consist of abnormalities in iron utilization, decrease in half-life of red blood cells, direct inhibition of hematopoiesis, and relative deficiency of erythropoietin [61].

Hospitalized patients feature acute or chronic inflammation caused by immune activation and occurring associated with anemia, ACD being the most common form found in these patients [62]. Under these conditions, erythropoiesis can be directly inhibited by an increase in the production of inflammatory cytokines inducing changes in iron homeostasis which could be characterized by reductions in both iron absorption and macrophage iron release [63]. Iron is a fundamental component of all living cells because iron is a cofactor for mitochondrial respiratory chain enzymes, the citric acid cycle, DNA synthesis, as well as an essential component for the transport of O_2_ through the hemoglobin and myoglobin. In addition, a sufficient amount of iron is important for immune preservation due to the fact of its role in promoting the growth of immune system cells, as the immune function and iron metabolism are widely linked.

Anemia of chronic disease is not considered a frequent cause of anemia in celiac patients; in fact, systemic inflammation, based on the increase in serum levels of acute phase proteins, is rare in CD patients, although gliadin-dependent activation of mononuclear cells of the mucous lamina propria causes an overproduction of proinflammatory cytokines such as interferon-γ (IFN-γ) and interleukin-6 (IL-6) [64,65]; both cytokines are mediators of ACD [66,67]. These proinflammatory cytokines are key factors in iron metabolism and in the development of ACD in celiac patients.

Thus, IL-6 inhibits the expression of the transferrin receptor mRNA, stimulates the synthesis of DMT-1, and is a mediator of hypoferremia in inflammation, which induces the synthesis of the hepcidin hormone regulating the iron export. An increase in hepcidin synthesis causes an increase in the degradation of ferroportin and the inhibition of iron release by the enterocyte, which leads to the alteration in the iron homeostasis associated with ACD [10,68]. IFN-γ stimulates ferritin transcription but at the same time inhibits its translation. IFN-γ also inhibits the transferrin receptor mRNA expression, which blocks the incorporation of iron mediated by the transferrin receptor, but increases the expression of DMT-1, thereby increasing the uptake and storage of ferrous iron. IFN-γ also decreases the mRNA of the transmembrane protein ferroportin, which exports iron to the outside of the cells. Therefore, IFN-γ favors iron retention within monocytes [52].

In this sense, some cytokines such as TNF-alpha, IL-1, and IL-10 are also released into circulation due to the inflammatory process [69]. These cytokines act on the liver and they contribute to the increase of hepcidin production, inhibiting the duodenal absorption of dietary iron. DMT-1 expression can also be induced by these cytokines. The net effect is the uptake of circulating iron in the reticuloendothelial system. In addition, IL-15 also seems to contribute to this pathway [70]. IL-15 is involved in the pathophysiology of CD and is partly responsible for sustained inflammation in active disease [71]. Taking into account all the above mentioned inflammatory pathways, CD could contribute to the development of de novo ACD. GFD is capable of reducing the oxidative state of patients with CD, although chronic inflammation persists even after two years of GFD. These patients showed a persistent high level of IFN-γ, IL-1α, interferon-inducible protein 10 (IP-10), and tumor necrosis factor beta (TNF-β) [23].

Bergamaschi et al. [72] studied anemia in patients with CD, reporting that ACD affected 17% of the subjects (11 out of 65 patients). Iron status parameters are similar in patients with ACD and those usually found during inflammatory processes, and an isolated iron deficiency or other pathogenic mechanisms could not be the explanation for their anemia. Their results reported a defective production of endogenous erythropoietin, in addition to changes in iron homeostasis, as a pathogenic mechanism of ACD. Other study conducted by Harper et al. [23] also confirmed that ACD can affect patients with CD and this fact is not completely unexpected, although these patients generally lack signs of systemic inflammation. Even though, the mean serum levels of inflammatory cytokines contributing to ACD (including IL-1β, IL-6, TNF-α, and IFN-γ) increased during active CD [73,74,75,76]. Although with a lower prevalence (3.9%), Berry et al. [77] also reported the presence of ACD in patients with CD.

In light of the observed studies and although ACD is not the most prevalent hematological disorder in patients with CD, it is necessary to take into account the pathogenesis of CD influence on its pathogenesis, given the role of iron in inflammatory signaling and in the turnover of epithelial cells.

## 5. Refractory Anemia to the Gluten-Free Diet

The etiology of persistent refractory anemia is multiple, and it must first be ruled out that it is due to the poor adherence to a GFD. Other causes of refractory anemia are chronic inflammation or anemia of chronic disorders, refractory celiac disease (RCD), the higher prevalence of the disease than expected by the involvement of other intestinal sections or the appearance of other comorbidities [77].

The first suggested finding is that it is a false refractoriness or persistence of anemia because the adherence to the treatment is not being conducted correctly. GFD is not easy to comply with nor is it generally well performed [78]. The traditional methods used to monitor the disease have poor performance, because, for example, with the serological method, for every six examinations we would detect the transgression in only one of them [79], besides presenting little correlation with villus atrophy [80]. The immunogenic gluten peptide in feces is postulated as a better tool for assessing diet adherence [81].

Celiac disease responds in the majority of patients on a GFD in a few weeks [13]. However, despite the correct adherence to a GFD, villous atrophy, malabsorption, and chronic intestinal inflammation persist in some patients for 12 months, which defines the RCD [82,83,84]. This can lead to persistence of symptoms and signs, including anemia. RCD is considered a rarity in pediatric age and, although its exact prevalence and incidence in adulthood is unknown, it is an uncommon condition [85]. Due to the poor response of the disease to treatment at this stage and its prognosis, it is important to correctly make the diagnosis [86], which is considered exclusion. The complete histological evaluation of the entire small intestine is needed for the diagnosis of refractoriness or complications [87].

In a recent study [88], mucosal involvement in patients with RCD was compared to patients with uncomplicated CD, showing that the involvement was greater in patients’ refractory to treatment, which may indicate that one of the causes of the persistence of symptoms is precisely the greatest extent of the disease. In a study [89] conducted in adult patients with CD and persistent IDA, 23% of patients showed lesions that were detected by video capsule endoscopy (VCE) of the small intestine. In another recent study conducted in pediatric CD patients [90], patients with anemia at diagnosis showed significantly larger histological lesions than CD patients without anemia; 92% of the patients recovered from the anemia after one year of adherence to a GFD. In patients with suspected RCD, especially type II, the performance of VCE is recommended [91]. Video capsule endoscopy is a relatively safe method with high sensitivity (approximately 89%) and specificity (approximately 95%) [92] to detect villus atrophy, and this could help differentiate RCD type I and II [88].

Furthermore, it is important to distinguish patients with uncomplicated CD from those with RCD, due to the risk of developing complications such as enteropathy associated with T-cell lymphoma (EATL), adenocarcinoma, jejunoileitis or B-cell lymphoma [93,94]. If left untreated, CD presents an increased risk of developing long-term tumors, especially of EATL and small bowel adenocarcinoma [95] compared to the general population. Enteropathy associated with T-cell lymphoma is sometimes diagnosed due to the signs and symptoms such as perforation, intestinal occlusion or bleeding, and persistent anemia, which may be an indicator of it. Likewise, ulcerative jejunoileitis is one of the phenotypic expressions of RCD. The characteristic symptom of this complication is abdominal pain, in relation to sub-occlusive symptoms, although the disease may present with hemorrhagic symptoms, perforation or protein-losing enteropathy due to the presence of inflammatory ulcers and strictures in the entire small intestine. In addition, it is associated with an increased risk of EATL [96].

The presence of other comorbidities, not always associated with CD itself, are linked to persistent symptoms once adherence to the GFD has been verified, such as microscopic colitis, irritable bowel syndrome, food allergies, motility disorders or collagen sprinkles [85]. The sprue collagen manifests itself in the form of refractoriness, and its occasional association with EATL has also been described [97]. The diagnosis is performed by biopsy and pathological analysis.

## 6. Conclusions and Future Perspectives

Celiac disease is a multisystemic disorder with different forms of clinical expression, from malabsorption with diarrhea, anemia, and growth retardation in children, to extraintestinal manifestations, such as those due to the fact of malabsorption and micronutrient deficiencies, including iron, folic acid, and vitamin B_12_. In fact, anemia may be the only clinical expression of the disease, and IDA is considered one of the most frequent extraintestinal clinical manifestations of CD. Celiac disease should be suspected in the presence of anemia without known etiology. Therefore, the determination of tissue anti-transglutaminase antibodies and anti-endomysial antibodies are indicated in these cases and, if positive, the performance of digestive endoscopy and intestinal biopsy is recommended.

Anemia in CD has a multifactorial pathogenesis and, although it is more frequently a consequence of iron deficiency, anemia can also be caused by deficiencies of folate or vitamin B_12_, as well as by blood loss or by its association with inflammatory bowel disease (IBD) or other associated diseases. The association between CD and IBD should be considered because the similarity of the symptoms could delay the diagnosis; the possibility of association among both pathologies should always be taken into account during the treatment of anemia in patients with IBD.

Vitamin B_12_ deficiency is common in CD and may be responsible for anemia and peripheral myeloneuropathy. Folate deficiency is a well-known cause of anemia in adults, but there is little information in children with CD. To date, it is still unknown if anemia is a symptom of the most typical CD in adult patients either by predisposition due to the age or because the biochemical and clinical manifestations take longer to appear.

Iron is a critical micronutrient whose deficiency in CD, in most cases, is a consequence of malabsorption secondary to the damage of the villi of the intestinal mucosa. However, iron deficiency in CD may also be a consequence of the reduced expression of different regulatory proteins. Alterations of iron absorption that could explain the inappropriate response to a GFD. It is known that the iron transporter DMT1 is positively regulated in CD to counteract iron malabsorption by villus atrophy, and that the risk of anemia in CD is related to the DMT-1 IVS + 44 AA genotype. A variant of this genotype can limit the overexpression of the transporter occurring normally prior to iron deficiency, being ineffective to counteract iron deficiency in the severe stage of the disease. Furthermore, the evaluation of the TMPRSS6 genotype, which influences iron metabolism through its effects on hepcidin, could be of clinical importance for the therapeutic management of iron supplementation, because a mutation can induce a poor response to iron therapy and predict the persistence of IDA despite iron treatment and GFD.

## Figures and Tables

**Table 1:** Etiology of anemia in celiac disease.

Cause	Incidence
Iron deficiency	12–69% (adults) 10–20% (children)
Folic acid deficiency	20–30%
Vitamin B_12_ deficiency	8–41%
Copper deficiency	Very low
Zinc deficiency	Very low *
Bad response to the gluten-free diet	23%
Medullary aplasia	Very low (12 cases)
Chronic disease	4–17%

* It has been reported that 50% of celiac patients have low serum levels at diagnosis, but it has not been related to celiac disease (CD).

**Table 2:** Characteristics of patients with aplastic anemia and celiac disease.

Study Reported	Grey-Davies [51]	Salmeron [53]	Maheswari [52]	Basu [54]	Badyal [55]	Omar [50]
Number of cases	3	5	1	1	1	1
Anemia diagnosis	Bone marrow biopsy	Bone marrow biopsy	Bone marrow biopsy	Bone marrow biopsy	Bone marrow biopsy	Bone marrow biopsy
Age (years)	23, 37, 43	Not reported	13	40	9	6
Intestinal biopsy	Villus atrophy	Villus atrophy	Villus atrophy	Villus atrophy	Not available	Villus atrophy
Treatment	GFD, corticotherapy, antithymocyte globulin, cyclosporine	GFD, antithymocyte globulin, cyclosporine. hematopoietic cell transplantation	GFD	GFD, corticotherapy, cyclosporine	GFD, corticotherapy, antithymocyte globulin	GFD

GFD; Gluten Free Diet.
